# Volumetric dried blood spots for determination of phosphatidylethanol: Validation of a liquid chromatography tandem masspectrometry method and clinical application

**DOI:** 10.1002/dta.3695

**Published:** 2024-04-25

**Authors:** Olof Beck, Miguel Barroso, Sigurd Hermansson, Cristopher Widén, Christer Wallin, Cecilia Nilsson‐Wallmark, Andrea de Bejczy

**Affiliations:** ^1^ Department of Clinical Neuroscience Karolinska Institutet Stockholm Sweden; ^2^ ABC Lab AB Stockholm Sweden; ^3^ Waters AB Solna Sweden; ^4^ Capitainer AB Solna Sweden; ^5^ Department of Neuroscience and Physiology, Sect. Psychiatry and Neurochemistry, Sahlgrenska Academy, Göteborg University and Beroendekliniken Sahlgrenska University Hospital Gothenburg Sweden

**Keywords:** alcohol biomarker, DBS, liquid chromatography mass spectrometry, microsampling, phosphatidylethanol

## Abstract

Phosphatidylethanol (PEth) measurement in whole blood samples is established as a specific alcohol biomarker with clinical and forensic applications. Establishment of dried blood spots (DBSs) as a specimen for PEth determination offers several advantages and was the focus of this work. A liquid chromatography tandem mass spectrometry method using a 96‐well format for sample preparation was developed and validated. PEth was extracted from DBSs by using isopropanol containing PEth‐d5 as internal standard. The blood sampling used a commercial volumetric DBS device having a phosholipase D inhibitor incorporated to stop continuous PEth formation. The method quantified PEth in the range of 0.05–10 μmol/L, with a bias and imprecision of less than 15%. In a clinical study (*n* = 25) using fingerprick blood, the volumetric device offered more precise quantifications (CV 4.6%) compared with the Whatman 903 Protein Saver card device (CV 16.6%). In another clinical study (*n* = 48), the use of dried venous and capillary blood, and liquid venous blood was compared under real‐life conditions with samples sent by postal service. The capillary and venous DBS samples gave identical results while the liquid blood gave slightly higher values. Calculation of elimination half‐life (PEth 16:0/18:1) in 31 cases based on two consecutive samples with 2–9 days in between gave results (mean 6.2 days) that agree with literature but several cases with values over 10 days. In conclusion, this study demonstrates that volumetric DBS is a valid specimen for determination of PEth blood concentrations, offering several advantages.

## Graphical abstract

Phosphatidylethanol, a specific alcohol biomarker, measured in dried blood spots was the focus of this work. A liquid chromatography tandem mass spectrometry method was developed, validated, and applied. The blood sampling used a volumetric DBS device. The volumetric device gave accurate and precise quantifications and the dried capillary blood gave the same results as that of venous blood.

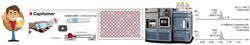

## 1 INTRODUCTION

Measurement of phosphatidylethanol (PEth) in venous whole blood has become established as an alcohol biomarker offering unique features.^1^, ^2^, ^3^ During its formation, PEth incorporates the ethanol molecule and becomes accumulated in cell membranes resulting in a biomarker with combined high specificity and sensitivity. PEth concentrations in whole blood is typically used to detect individuals with repeated frequent drinking over time, as well as for monitoring its elimination after abstinence during treatment for alcohol use disorder (AUD). The slow decline of whole blood PEth, with an elimination half‐life of almost a week, is the foundation for its usability as a cumulative biomarker of recent alcohol drinking.^1^ A lower limit of 0.03–0.05 μmol/L (~20 ng/mL) for “social” drinking and a higher limit of about 0.3 μmol/L (~200 ng/mL) for “excessive unhealthy” drinking is commonly accepted.^3^, ^4^, ^5^

In detecting risky drinking habits and in monitoring treatment effects, a reliable biomarker for alcohol intake is of utmost importance. PEth is superior to traditional indirect biomarkers such as carbohydrate deficient transferrin and gamma‐glutamyl transferase, in accurately mirroring recent alcohol intake^6^ and also superior to subjective reporting, which suffers from limitations such as recall bias.^7^ An easy‐to‐use blood biomarker to correctly assess alcohol consumption is an invaluable asset in alcohol treatment strategies, as well as in alcohol research studies.

The need for collecting venous blood samples may be a limiting factor in many instances. Dried blood spots (DBSs) based on capillary blood sampling offer a solution and have been applied for measuring PEth concentrations.^8^, ^9^, ^10^, ^11^, ^12^ This makes the PEth biomarker available in a broader array of settings, for example, treatment centers. Another benefit is the possibility to prohibit post‐sampling formation of PEth by inhibiting the activity of the phospholipase D (PLD, EC 3.1.4.4) involved in the formation and decomposition of PEth.^13^, ^14^ The PLD inhibition is considered crucial for enabling valid sampling,^15^ leading to the development of a DBS device having such an inhibitor incorporated in the filter disc.^16^

The aim of the current work was to develop and validate a method for automated high‐throughput application and, in addition, to perform two clinical studies to test the reliability of the method. The use of the volumetric device was compared with a standard DBS device to estimate the additional accuracy offered. In a second clinical study, the use of venous blood was compared with capillary blood collected as DBS.

## 2 EXPERIMENTAL

### 2.1 Chemicals and material

PEth (16:0/18:1) and PEth‐d5 were from Cerilliant Corporation (Round Rock, TX, USA). PEth 18:1/16:0 was from Merck KGaA (Darmstadt, Germany). Chemicals and water used were from commercial sources and were of LC–MS purity.

The DBS filter paper cards for microsampling of blood were the Whatman 903 Protein Saver card (GE HealthCare Ltd, Cardiff, UK) and the Capitainer®B Vanadate (qDBSV) quantitative DBS device (Capitainer AB, Solna, Sweden).

### 2.2 Blood samples

The blood specimens used for this study were de‐identified surplus volumes of fresh venous whole blood samples sent for routine analysis or from healthy volunteers and in‐ward patients with AUD after informed consent. Venous blood was collected in EDTA tubes and stored at 4°C before and after postal delivery for maximum 1 week or directly kept frozen at −20°C until analysis up to a maximum of 3 months. Prepared DBS standards and quality control samples were stored at −80°C up to a maximum of 1 year.

Blood containing ethanol was prepared by adding a 60% ethanol solution and immediately used for the experiments. Prepared DBS samples were put in freezer directly or left to dry at room temperature overnight.

The clinical studies were approved by the Swedish Ethical Review Authority (Dnr 2022‐01823‐01) and by the Ethics Committee at the Karolinska University Hospital (No. 2013/341‐31/4).

### 2.3 LC–MS/MS measurement of PEth 16:0/18:1

Measurement of PEth was done by a laboratory developed LC–MS/MS method. The 6 mm diameter filter was taken out from the volumetric device, or a 6 mm diameter punch from standard device, and placed in a well of a 96‐well Sirocco Protein Extraction Plate (Waters Corporation, Milford, MA, USA) that was placed on top of a 2 mL 96‐well collection plate. The filter was wetted by adding 20 μL of pure water. After a minimum time of 5 min, 200 μL of isopropanol (IPA) containing PEth‐d5 (5.1 pmol) was added. The plates were gently shaken (700 rpm) for 30 min and centrifuged at 2000 × *g* for 5 min. The extraction and centrifugation steps were repeated using 200 μL IPA. Finally, the collection plate was covered with a silicon cap mat and placed in the autosampler.

Standards were prepared from discs prepared from blank blood, and PEth in IPA was added after the extraction solvent (first step) was added to the well. The applied measuring range was 0.05–10 μmol/L. Quality control samples were prepared by applying selected authentic blood with assigned concentrations (~0.05, 0.30, and 1.5 μmol/L) onto qDBSV devices. External quality samples were from EQUALIS AB (Uppsala, Sweden) and were obtained as liquid blood that was applied on qDBSV cards manually before analysis.

The LC–MS/MS instrument was a Waters Xevo TQ‐XS mass spectrometer equipped with a UniSpray ion source, an Acquity UPLC® I‐CLASS, and an Acquity UPLC® Sample Organizer (Waters Corporation). The system was controlled with the MassLynx software. Instrumental settings were cone voltage 60 V, collision energy 30 eV, and ion source and desolvation temperatures were 150°C and 600°C, respectively.

The chromatographic system consisted of an Acquity Premier BEH C18 column, 1.7 μm, 2.1 × 50 mm (Waters Corporation), with a column temperature of 65°C and a flow rate of 0.6 mL/min. Mobile phase A was pure water with 20% acetonitrile (AcCN) (v/v) and 10 mmol/L ammonium acetate, and mobile phase B was methanol (MeOH) with 20% AcCN (v/v). The gradient elution program started at 13% A followed by a linear gradient to 100% B from 0 to 2.5 min and finally back to 13% A. The time between injections (volume 1.0 μL) was 3.0 min. The wash solvent was a mix of MeOH, AcCN, IPA, and pure water (25% each, v/v) with 2% formic acid (v/v), and the purge solvent was 20% MeOH in pure water (v/v).

The precursor ions (*m/z*) of the monitored transitions were 701.3 for PEth and 706.3 for PEth‐d5, and the quantifier ion was 281.2 and qualifier ion 255.2 for both compounds. Identification criteria were a relative retention time (analyte/internal standard) within ±0.5% and an ion ratio within ±20%.

### 2.4 Method validation

Calibration curves were run on six different days in the concentration range of 0.036–8.53 μmol/L at eight different levels. The imprecision in measurement was studied by the running QC samples with four replicates on five different days. Calculation of imprecision was done using the CLSI template.^17^

The LLOQ was established by analyzing 10 repetitions of a 0.025 μmol/L standard.

LOD and background peaks in blank blood were studied using reference blank blood and blank qDBSV discs.

Extraction recovery was studied by doing three repetitive extractions and by comparing extraction of PEth in DBS and by adding PEth to blank blood extracts.

Matrix effect was studied by post column infusion of a blank blood extract and by an addition experiment using blank blood matrix (*n* = 4) comparing the response with and without DBS matrix.

Stability of PEth in DBS format was studied for 1 month at RT, +4°C, −20°C, and −80°C.

Accuracy of the measurements was studied by comparing with an accredited reference lab and by using samples from the EQUALIS proficiency testing program. In both cases, supplied liquid blood was applied on qDBSV cards prior to analysis.

The effect of drying was studied by applying venous blood at qDBSV devices and let dry at room temperature for up to 24 h.

### 2.5 Clinical Study 1

Twenty‐five healthy volunteers were recruited after giving self‐reported information regarding regular alcohol drinking. The individuals were instructed to self‐sample finger blood using two qDBSV devices (four spots) and one Whatman 903 Protein Saver card giving five spots. The blood sampling was done after washing the finger with an isopropanol swab. The lancet used was a Becton Dickinson Microtainer lancet blue (Becton, Dickinson and Company, Franklin Lakes, NJ). The first drop of blood was discarded.

### 2.6 Clinical Study 2

A second study recruited AUD in‐ward patients during alcohol withdrawal treatment. Sobriety was assessed by breath alcohol concentration before signed informed consent. Forty‐eight patients were included in the study. One EDTA test tube of venous blood and capillary blood by fingerprick on a qDBSV device were collected from each subject. Additionally, a subsample of the venous blood was dropped on a qDBSV device. When possible, a second sample was taken from each patient within 2–9 days. All samples from Subjects 1–32 were directly put in storage at −20°C.

In the second phase of the study (Patients 33–48), all samples were sent directly by postal mail to the laboratory to mimic real‐life conditions. The analysis was performed directly after arrival. Surplus liquid venous blood was then stored at +4°C and surplus qDBSV discs at −80°C.

The aliquot of liquid blood used for measuring PEth concentrations was prepared by using the volumetric device in order to assure the same volume of blood for analysis. The blood collected on the disc was used directly without drying.

## 3 RESULTS

### 3.1 Method design and validation

**FIGURE 1:**
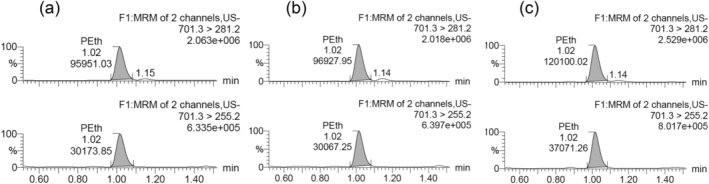
Representative chromatograms from the selected reaction monitoring liquid chromatography tandem mass spectrometry analysis of PEth in three samples from the same patient: (a) capillary blood as dried blood spot, (b) venous blood as dried blood spot, and (c) liquid venous blood.

The method was designed for high‐throughput application, and therefore, the 96‐well format was chosen. The use of a filter plate gave extracts clean from particles prone to cause chromatographic back pressure over time. The two‐step extraction was necessary to provide a high recovery. The extraction time appeared not critical because the recovery did not increase after 5 min shaking time. The LC–MS/MS procedure and conditions were provided from the Waters application laboratory and are used by several laboratories. Representative chromatograms are shown in Figure 1.

The calibration curve was not linear over the whole range, the slope tended to level off, and therefore, it was divided in two ranges, that is, below 1 μmol/L and entire range. Below 1 μmol/L, the linear regression analysis gave a coefficient of determination (*r* ^2^) that always was ≥0.9995 and in the whole range >0.9985.

The results for imprecision of the measurements are shown in Table 1. The total imprecision was always below 9%. At 50% (0.025 μmol/L) of the lower limit of the measuring range the CV at that concentration level was 12.5% (*n* = 10). The limit of detection was estimated to 0.002 μmol/L based on a signal‐to‐noise ratio of 3 in blank blood for the weakest transition (*m/z* 701.5 → 255.2).

**TABLE 1:** Results for quality control samples analyzed in quadruplicate for 5 days.

	Assigned concentration (μmol/L)	Intra‐day CV (%)	Total CV (%)	*n*
QCL	0.062	5.2	8.9	20
QCM	0.22	7.0	6.8	20
QCH	1.15	5.8	5.4	20
QCHH	5.85	4.3	4.5	20

Method comparison was used to document accuracy. Twenty negative reference blood samples (<0.05 μmol/L) all came out as <0.015 μmol/L. Twenty reference samples in the interval 0.056–3.58 μmol/L gave a mean bias of −2.3%. The regression analysis gave a slope of 1.05 and a coefficient of determination of 0.986. Four reference samples from the EQUALIS proficiency program having assigned concentrations of 0.13, 0.54, 0.63, and 1.63 μmol/L gave the following results (mean of duplicate analysis): 0.13, 0.53, 0.63, and 1.58, respectively.

The matrix effect from blood was first studied by post column infusion with no effect observed. In the addition experiment, a reduction of response of 16% was observed. Extraction recovery was studied by repetitive extraction, and after two extractions with IPA, the recovery was >98%. When estimating recovery by comparing with a reference sample having PEth added after extraction, the recovery was 101 ± 4%.

Stability was studied using 20 authentic blood specimens. Cards stored at −80°C were used as reference. The following results for RT, +4°C, and −20°C were obtained: 92.3 ± 8.6%, 92.2 ± 6.3%, and 92.1 ± 6.5%. The concentrations obtained for the −80°C stored reference samples agreed with those determined before storage with a mean bias of −2.3%.

A blank blood was fortified with 1‰ ethanol, and the formation of PEth after the drying process was examined. Without ethanol the estimated (calculated from ion ratio using the slope from the calibration equation) mean PEth concentration was 0.0034 ± 0.0003 μmol/L (*n* = 4) which increased to 0.0052 ± 0.0011 μmol/L (*n* = 40) with ethanol present.

The two isomers of PEth (16:0/18:1 and 18:1/16:0) were investigated and were found to co‐elute in the system used. Also, with a much slower gradient the two isomers could not be separated chromatographically. However, the isomers had very different ratios between the response for the two transitions (quantifier/qualifier) monitored. For the 16:0/18:1 isomer, the ratio was 3.4 while for 18:1/16:0, it was 0.34 when studied in pure solutions. In authentic blood samples containing PEth, the ratio was 3.27 ± 0.11 (*n* = 20), while for calibrator standards, it was 3.53 ± 0.09 (*n* = 14).

In one experiment, liquid blood was applied on qDBSV devices and analyzed without and with drying overnight. The ratio between the dried and not dried specimens was 1.00 ± 0.096 (*n* = 12). The effect of drying was also studied over time with drying times 0–24 h (*n* = 20). The result is presented in Table 2. At the 3 h drying time and beyond, no difference to not dried specimens was observed.

**TABLE 2:** Drying time experiment.

Drying time (h)	Mean % compared with direct	SD	*n*
1	90.4	5.5	19
3	97.0	5.2	19
6	97.1	6.1	20
9	98.7	5.5	19
24	97.5	6.5	20

*Note*: Ten different venous blood samples containing PEth were applied as dried blood spots. Samples from each time point was analyzed in duplicate when possible.

### 3.2 Clinical Study 1

In the healthy volunteer study, three out of the 25 participants had PEth concentrations below 0.05 μmol/L and were omitted from the data analysis. Another two participants did not provide the standard DBS specimen. Out of the 22 cases that provided qDBSV devices, four provided only three discs and six provided only two. The calculated mean imprecision was estimated to 4.57% (*n* = 22), while for the Protein Saver card specimens, the mean imprecision was 10.2% (*n* = 20). The calculated mean PEth concentration for each case was used to set the volume of blood collected on the punch from the Protein Saver card by using the qDBSV values as reference for 10 μL. The range was 7.58–13.0 μL with a mean of 10.84 ± 1.43 μL. When taking this additional uncertainty into consideration, the total uncertainty in measurement (CV) using the Protein Saver card was calculated to 16.6%.

### 3.3 Clinical Study 2

Forty‐eight patients were included in the study and provided venous and capillary blood samples at a first occasion. A second sample was obtained from 33 patients. One patient had an initial PEth concentration <0.05 μmol/L and was omitted from the data analysis. The concentration range calculated from the total of 98 capillary qDBSV specimens was 0.32–6.41 μmol/L.

In the first phase of the study (Subjects 1–32), with specimens stored in freezer immediately after sampling, the concentration ratio between capillary and venous blood collected as qDBSV was 0.99 ± 0.10 (*n* = 32). Linear regression analysis gave a slope of 0.98 and *r* ^2^ of 0.963. The ratios between DBS specimens and liquid venous blood were 0.88 ± 0.08 for capillary qDBSV and 0.87 ± 0.13 for venous qDBSV. In the second phase of the study (Subjects 33–48), with samples sent to lab with postal mail (*n* = 15), the ratio between capillary and venous blood DBS was 1.03 ± 0.06, and the ratios between DBS specimens and liquid venous blood were 0.87 ± 0.10 for capillary qDBSV and 0.90 ± 0.10 for venous qDBSV.

The elimination half‐life of PEth was calculated in the cases that provided two specimens based on first order kinetics (*n* = 31). The range of half‐life was 2.6 to 15 days with a mean of 7.2 and median of 6.4 days.

## 4 DISCUSSION

An LC–MS/MS method for measuring PEth in volumetric DBS specimens was successfully developed and validated. The method provides precise and accurate results according to the method validation, thereby providing an alternative specimen for PEth determination that does not require sampling at a professional health setting. PEth has been measured using DBS specimens before.^8^, ^9^, ^10^, ^11^, ^12^ The unique feature of the device employed in this study is the volumetric design that provides a precise and accurate blood volume for analysis independent of the hematocrit value.^18^, ^19^ In addition, the presence of a PLD inhibitor ensures a correct measurement of PEth even when ethanol is present in the blood.^16^

Post‐sampling formation of PEth occurs when ethanol is present in the blood, which usually is an unknown parameter. It has been noted that in clinical routine application, several percent of samples arriving at laboratory for PEth analysis contains ethanol.^20^, ^21^ It has been advocated that a PLD inhibitor needs to be added for making an accurate measurement of PEth concentrations at the time of sampling.^15^ It has also been advocated that the post‐sampling formation can be neglected,^22^ but this conception is based on a very limited number of samples investigated.^20^ Because both the ethanol concentration and PLD activity are unknown factors, the risk of doing erroneous determinations of PEth must be handled with great concern.

In Clinical Study 1, the objective was to estimate the uncertainty associated with the use of a standard DBS device where the subsample is punched out and used for analysis, which has been a subject for study before for other analytes.^23^, ^24^ The value for the blood volume from the 6 mm diameter punch was calculated to 10.8 μL. However, under conditions without the possibility to calibrate the blood volume value, the uncertainty will be higher than the 16.6% calculated in this study. In the other studies using the same filter paper, the volume of blood punched out with a 6 mm diameter punch was reported to be 8.7 ± 1.9^23^ and 12.84 μL.^24^ This additional uncertainty adds to the total one and might result in an unacceptable level of uncertainty, similar to what has been advocated for the monitoring of phenylalanine in patients suffering from phenylketonuria.^25^ A method for PEth measurement based on standard DBS has been published in Nature protocols without considering these additional factors of uncertainty.^26^

In Clinical Study 2 involving AUD patients, it was confirmed that capillary blood is a valid specimen for PEth determination.^8^, ^9^, ^10^, ^11^, ^12^ However, about 10% higher concentration values were measured when using the liquid venous blood, both when the specimens were stored in freezer immediately and when they were sent to the laboratory by postal mail. In order to investigate this observation further, which was unexpected based on the method validation results, additional experiments were carried out. A time‐dependent effect during drying on the measured PEth concentration was observed in the sense that the PEth concentration values dropped initially (1 h) and then recovered (Table 2). This observation could still not explain the difference between dried and wet blood specimens but should be considered in the future.

Elimination half‐life of PEth after alcohol abstinence has been documented in both in‐ and out‐patient settings with and without rigorous control regarding continued alcohol intake.^27^, ^28^ The mean and median elimination half‐life of PEth (16:0/18:1) is reported to be 6–8 days and with a range of 3–10 days. Our result from the 31 in‐patients under strict control agrees with these reports despite the limitations of our calculations to be based on only two consecutive samplings. Data for PEth elimination half‐life in healthy volunteers after a single dose of ethanol agree with these results.^29^

Another important issue to consider for accurate PEth measurement, first raised by Luginbühl et al., is the occurrence of two positional isomers (16:0/18:1 vs. 18:1/16:0).^30^ This has relevance for both the selection of reference material and for what is to be measured in the biological samples. The PEth reference material that has the correct isomer was used in our method for quantification. We could verify that the 18:1/16:0 isomer co‐elutes chromatographically and gives a much different relative detector response for the two ion transitions monitored. We could present data from 20 cases investigated showing that a minor part of the natural occurring PEth is the 18:1/16:0 isomer.

## 5 CONCLUSIONS

This study demonstrates that volumetric DBS is a valid specimen for determination of PEth blood concentrations, offering several advantages as a method for assessing alcohol consumption.

## CONFLICT OF INTEREST STATEMENT

One author (O.B.) is involved in the Capitainer AB company as a co‐founder. Andrea de Bejczy is founder and co‐owner of Sobrera Pharma AB. The other authors declare no conflicts of interest.

## References

[1] Isaksson A , Walther L , Hansson T , Andersson A , Alling C . Phosphatidylethanol in blood (B‐PEth): a marker for alcohol use and abuse. Drug Test Anal. 2011;3(4):195‐200. doi:10.1002/dta.278 21438164

[2] Viel G , Boscolo‐Berto R , Cecchetto G , Fais P , Nalesso A , Ferrara SD . Phosphatidylethanol in blood as a marker of chronic alcohol use: a systematic review and meta‐analysis. Int J Mol Sci. 2012;13(11):14788‐14812. doi:10.3390/ijms131114788 23203094 PMC3509610

[3] Ulwelling W , Smith K . The PEth blood test in the security environment: what it is; why it is important; and interpretative guidelines. J Forensic Sci. 2018;63(6):1634‐1640. doi:10.1111/1556-4029.13874 30005144

[4] Helander A , Hansson T . Nationell harmonisering av alkoholmarkören PEth [National harmonization of the alcohol biomarker PEth]. Lakartidningen. 2013;110(39–40):1747‐1748. Swedish. PMID: 24245431. 24245431

[5] Helander A , Hermansson U , Beck O . Dose‐response characteristics of the alcohol biomarker phosphatidylethanol (PEth)—a study of outpatients in treatment for reduced drinking. Alcohol Alcohol. 2019;54(6):567‐573. doi:10.1093/alcalc/agz064 31529064

[6] Walther L , de Bejczy A , Löf E , et al. Phosphatidylethanol is superior to carbohydrate‐deficient transferrin and γ‐glutamyltransferase as an alcohol marker and is a reliable estimate of alcohol consumption level. Alcohol Clin Exp Res. 2015;39(11):2200‐2208. doi:10.1111/acer.12883 26503066

[7] Stevens AK , Sokolovsky AW , Treloar Padovano H , White HR , Jackson KM . Heaviness of alcohol use, alcohol problems, and subjective intoxication predict discrepant drinking reports in daily life. Alcohol Clin Exp Res. 2020;44(7):1468‐1478. doi:10.1111/acer.14362 32530512 PMC7572532

[8] Faller A , Richter B , Kluge M , et al. LC‐MS/MS analysis of phosphatidylethanol in dried blood spots versus conventional blood specimens. Anal Bioanal Chem. 2011;401(4):1163‐1166. doi:10.1007/s00216-011-5221-y 21743983

[9] Beck O , Kenan Modén N , Seferaj S , Lenk G , Helander A . Study of measurement of the alcohol biomarker phosphatidylethanol (PEth) in dried blood spot (DBS) samples and application of a volumetric DBS device. Clin Chim Acta. 2018;479:38‐42. doi:10.1016/j.cca.2018.01.008 29309773

[10] Jones J , Jones M , Plate C , Lewis D . The detection of 1‐palmitoyl‐2‐oleoyl‐sn‐glycero‐3‐phosphoethanol in human dried blood samples. Anal Methods. 2011;3(5):1101‐1106. doi:10.1039/C0AY00636J

[11] Bakhireva LN , Savich RD , Raisch DW , et al. The feasibility and cost of neonatal screening for prenatal alcohol exposure by measuring phosphatidylethanol in dried blood spots. Alcohol Clin Exp Res. 2013;37(6):1008‐1015. doi:10.1111/acer 23421919 PMC3661684

[12] Kummer N , Ingels AS , Wille SM , et al. Quantification of phosphatidylethanol 16:0/18:1, 18:1/18:1, and 16:0/16:0 in venous blood and venous and capillary dried blood spots from patients in alcohol withdrawal and control volunteers. Anal Bioanal Chem. 2016;408(3):825‐838. doi:10.1007/s00216-015-9169-1 26597914

[13] Aradóttir S , Seidl S , Wurst FM , Jönsson BA , Alling C . Phosphatidylethanol in human organs and blood: a study on autopsy material and influences by storage conditions. Alcohol Clin Exp Res. 2004;28(11):1718‐1723. doi:10.1097/01.alc.0000145687.41646.e5 15547459

[14] Faller A , Richter B , Kluge M , Koenig P , Seitz HK , Skopp G . Stability of phosphatidylethanol species in spiked and authentic whole blood and matching dried blood spots. Int J Leg Med. 2013;127(3):603‐610. doi:10.1007/s00414-012-0799-y 23208617

[15] Schröck A , Henzi A , Bütikofer P , König S , Weinmann W . Determination of the formation rate of phosphatidylethanol by phospholipase D (PLD) in blood and test of two selective PLD inhibitors. Alcohol. 2018;73:1‐7. doi:10.1016/j.alcohol.2018.03.003 30103144

[16] Beck O , Mellring M , Löwbeer C , Seferaj S , Helander A . Measurement of the alcohol biomarker phosphatidylethanol (PEth) in dried blood spots and venous blood‐importance of inhibition of post‐sampling formation from ethanol. Anal Bioanal Chem. 2021;413(22):5601‐5606. doi:10.1007/s00216-021-03211-z 33590314 PMC8410693

[17] CLSI . (Ed). User Verification of Precision and Estimation of Bias; Approved Guideline. 3rd ed CLSI document EP215‐A3. Clinical and Laboratory Standards Institute; 2014. https://clsi.org/standards/products/method-evaluation/documents/ep15/

[18] Spooner N , Olatunji A , Webbley K . Investigation of the effect of blood hematocrit and lipid content on the blood volume deposited by a disposable dried blood spot collection device. J Pharm Biomed Anal. 2018;5(149):419‐424. doi:10.1016/j.jpba.2017.11.036 29154197

[19] Velghe S , Stove CP . Evaluation of the Capitainer‐B microfluidic device as a new hematocrit‐independent alternative for dried blood spot collection. Anal Chem. 2018;90(21):12893‐12899. doi:10.1021/acs.analchem.8b03512 30256092

[20] Isaksson A , Walther L , Hansson T , Andersson A , Stenton J , Blomgren A . High‐throughput LC‐MS/MS method for determination of the alcohol use biomarker phosphatidylethanol in clinical samples by use of a simple automated extraction procedure‐preanalytical and analytical conditions. J Appl Lab Med. 2018;2(6):880‐892. doi:10.1373/jalm.2017.024828 33636821

[21] Neumann J , Beck O , Helander A , Böttcher M . Performance of PEth compared with other alcohol biomarkers in subjects presenting for occupational and pre‐employment medical examination. Alcohol Alcohol. 2020;55(4):401‐408. doi:10.1093/alcalc/agaa027 32363383 PMC7338721

[22] Helander A , Hansson T . The alcohol biomarker phosphatidylethanol (PEth)—test performance and experiences from routine analysis and external quality assessment. Scand J Clin Lab Invest. 2023;83(6):424‐431. doi:10.1080/00365513.2023.2253734 37697976

[23] Hewawasam E , Liu G , Jeffery D , Gibson R , Muhlhausler B . Estimation of the volume of blood in a small disc punchedfrom a dried blood spot card. Eur J Lipid Sci Technol. 2018;120(3):1700362. doi:10.1002/ejlt.201700362

[24] Moat SJ , Zelek WM , Carne E , et al. Development of a high‐throughput SARS‐CoV‐2 antibody testing pathway using dried blood spot specimens. Ann Clin Biochem. 2021;58(2):123‐131. doi:10.1177/0004563220981106 33269949 PMC7844389

[25] Moat SJ , Schulenburg‐Brand D , Lemonde H , et al. Performance of laboratory tests used to measure blood phenylalanine for the monitoring of patients with phenylketonuria. J Inherit Metab Dis. 2020;43(2):179‐188. doi:10.1002/jimd.12163 31433494 PMC7957320

[26] Luginbühl M , Stöth F , Schröck A , Gaugler S , Weinmann W . Quantitative determination of phosphatidylethanol in dried blood spots for monitoring alcohol abstinence. Nat Protoc. 2021;16(1):283‐308. doi:10.1038/s41596-020-00416-x 33288956

[27] Helander A , Péter O , Zheng Y . Monitoring of the alcohol biomarkers PEth, CDT and EtG/EtS in an outpatient treatment setting. Alcohol Alcohol. 2012;47(5):552‐557. doi:10.1093/alcalc/ags065 22691387

[28] Helander A , Böttcher M , Dahmen N , Beck O . Elimination characteristics of the alcohol biomarker phosphatidylethanol (PEth) in blood during alcohol detoxification. Alcohol Alcohol. 2019;54(3):251‐257. doi:10.1093/alcalc/agz027 30968936 PMC7011165

[29] Javors MA , Hill‐Kapturczak N , Roache JD , Karns‐Wright TE , Dougherty DM . Characterization of the pharmacokinetics of phosphatidylethanol 16:0/18:1 and 16:0/18:2 in human whole blood after alcohol consumption in a clinical laboratory study. Alcohol Clin Exp Res. 2016;40(6):1228‐1234. doi:10.1111/acer.13062 27130527 PMC4939838

[30] Luginbühl M , Young RSE , Stoeth F , Weinmann W , Blanksby SJ , Gaugler S . Variation in the relative isomer abundance of synthetic and biologically derived phosphatidylethanols and its consequences for reliable quantification. J Anal Toxicol. 2021;45(1):76‐83. doi:10.1093/jat/bkaa034 32248226

